# Characteristics of Bacterial Biofilm Formation in Nasolacrimal Silicone Tubes Post-dacryocystorhinostomy

**DOI:** 10.7759/cureus.56112

**Published:** 2024-03-13

**Authors:** Şule Berk Ergun, Elif G Has, Nefise Akçelik, Mustafa Akçelik

**Affiliations:** 1 Department of Ophthalmology, Ankara Bilkent City Hospital, Ankara, TUR; 2 Department of Biology, Ankara University Faculty of Science, Ankara, TUR; 3 Biotechnology Institute, Ankara University, Ankara, TUR

**Keywords:** streptococcus, staphylococcus, salmonella, pseudomonas, klebsiella, dacryocystorhinostomy, biofilm

## Abstract

Purpose: To examine the biofilm formation characteristics of bacteria identified at the genus level in samples obtained from silicone tubes after dacryocystorhinostomy surgery.

Methods: In the study involving consecutive patients who underwent dacryocystorhinostomy surgery at Ankara Bilkent City Hospital and whose silicone tubes were removed six months after surgery, between January 2023 and May 2023; the tubes were placed in glycerol-PBS (phosphate buffered saline) solution and cultured on descriptive selective media at the genus level. The biofilm-forming properties of the obtained isolates were examined in solid-air and liquid-air interphases. *Salmonella* Typhimurium ATCC SL1344 strain was used as the control bacterium.

Results: As a result of the analysis of the samples taken from the patients, *Pseudomonas* spp. was identified in three of the samples, *Staphylococcus* spp. in five of the samples, and* Streptococcus* spp. in one of the samples. Among these samples, except for the bacteria identified in samples one and five, the rest were found to be strong biofilm producers. In all strong biofilm producers, the maximum biofilm production time was determined as 72 h and the incubation temperature was 37°C. The presence of cellulose and amyloid proteins in biofilm matrix structures is identified. Swimming and swarming motilities were observed in all bacterial samples.

Conclusion: Since biofilms are considered potential factors in the pathogenesis of infectious and inflammatory diseases, they are a subject that needs to be thoroughly investigated. In our study, although there were no clinical infections in any of the patients, biofilm formation was detected in the patient samples. The fact that the bacteria exhibited moderate to strong biofilm formation characteristics suggests that these microorganisms could be persistent infectious agents.

## Introduction

External dacryocystorhinostomy (DCR) is a widely accepted and commonly employed, well-established treatment method for nasolacrimal duct obstruction. It facilitates direct tear drainage by creating a surgical bypass between the lacrimal sac and nasal cavity [1]. It consistently achieves success rates exceeding 90% [2].

Numerous studies on concurrent silicone intubation during DCR have concluded that it is a safe and efficient approach for treating nasolacrimal duct obstruction [3,4]. Following these findings, the majority of surgeons have adopted the routine use of silicone tubes in all DCR procedures to enhance outcomes. Nonetheless, infection of the silicone tube can lead to postoperative failure [5].

In nature, bacteria adhere to various abiotic or biotic surfaces and establish communities characterized by differentiation and interaction, commonly referred to as "biofilms" [6].

Biofilms developed on silicone tubes have the potential to trigger persistent and chronic bacterial infections. They may instigate a chronic inflammatory response and ongoing infection by releasing microorganisms into the body. Furthermore, they can contribute to increased bacterial resistance and provoke a sustained inflammatory reaction at the biofilm site [7-9].

Although there have been studies investigating biofilm formation on lacrimal stents, this topic warrants detailed investigation, and their actual clinical implications are controversial [10-13].

The aim of this study is to contribute to the literature by examining the biofilm formation characteristics of bacteria identified at the genus level in samples obtained from silicone tubes removed from patients after DCR surgery.

## Materials and methods

Clinical samples were obtained from the Ophthalmology Clinic of Ankara Bilkent City Hospital. The study protocol was approved (No. E1-23-4566) by the institutional ethics committee of the Ankara Bilkent City Hospital and adhered to the tenets of the Declaration of Helsinki. Silicone tubes removed six months later from patients who underwent DCR surgery for nasolacrimal duct obstruction without a history of a dacryocystitis between January 2023 and May 2023 were then sterilized in Eppendorfs containing glycerol-PBS (phosphate buffered saline), and each patient was numbered consecutively. Selective media were used to determine the bacteria in each sample at the genus level. *Salmonella* Typhimurium ATCC SL1344 (SL1344) strain, which is known to produce biofilm, obtained from Ankara University Prokaryotic Genetics Laboratory, was used as the control in the studies. Mannitol Salt Agar (MSA), HiFluoro Pseudomonas Agar (HiFPA), Klebsiella Blue Agar (KBA), and Streptococcus Selective Agar (SSA) were used for the identification of *Staphylococcus*, *Pseudomonas*, *Klebsiella*,* *and *Streptococcus* spp. in the samples obtained from the patients, respectively. Tryptic Soy Agar (TSA) and Tryptic Soy Broth (TSB) were used as common media in the experiments for bacteria determined at the genus level. Active cultures were stocked at -20°C and -80°C in appropriate media containing 60% glycerol.

Biofilm assays and cellulose production

In order to determine the amount of biofilm production of clinical isolates on polystyrene surface, bacteria were inoculated into 5 mL TSB medium at 1% and activated overnight at 37°C. The optical density (OD) of the active cultures was adjusted to 0.2 ABS at 595 nm. In 96-well microtiter plates, 100 µL of TSB was added to each well and 30 µL of the bacterial suspensions with adjusted ODs were added in three parallel wells for each group and each hour. Only TSB was added to the control well. At the end of the incubation period, the wells were aspirated and washed three times with PBS to remove the medium and planktonic cells that did not adhere to the surface. Then 130 µL of 95% methanol was added to the wells and incubated for 30 minutes. At the end of the time, methanol was removed from the medium and allowed to dry for five minutes under room conditions. 130 µL of 1% crystal violet (stains only adherent biofilm cells) was added to the wells and incubated for 30 minutes. At the end of the incubation period, the wells were washed three times with distilled water and 130 µL of 33% glacial acetic acid was added to each well to dissolve the stain. After the crystal violet was completely dissolved, the amount of biofilm production was determined according to the optical densities by optical measurements at 595 nm in the ELISA (enzyme-linked immunosorbent assay) device [14]. The results of biofilm measurements were calculated by subtracting the mean of the OD values of the control wells (wells containing TSB only) from the mean of the optical density (OD) values from all three parallels. At the same time, the strains were categorized based on their "cut-off" values to be evaluated in terms of their biofilm production. This evaluation assessed the biofilm-forming capacity of the strains as "non-producer", "weak producer", " medium producer," and "strong producer" (Table 1).

**Table 1:** “Cut off” conversions used in the evaluation of biofilm production capacities. OD: optical density, ODc: optical density of the control group.

OD ≤ ODc	non-producer
ODc < OD ≤ 2xODc	weak producer
2xODc < OD ≤ 4xODc	medium producer
4xODc < OD	strong producer

10 µL of overnight active cultures were injected into the center of TSA petri plates with 40 μg/mL Congo red in order to identify the biofilm morphotypes. Petri dishes were incubated at 20°C for three days. At the end of the incubation period, biofilm forms were visually examined under a stereo microscope (Leica, Germany). The study was carried out in three parallel and two replicates [15].

For the determination of cellulose production, the main component of biofilm structures, overnight active cultures were seeded on TSA petri dishes containing 20 μg/mL Calcofluor. The plates were incubated at 20°C for three days. At the end of the incubation period, cellulose production was quantitatively evaluated under 366 nm UV light (the higher the fluorescence intensity, the higher the cellulose production) [16,17].

To study biofilm formations (pellicles) in the liquid-air interface phase, 500 µL of overnight active cultures were inoculated into 4500 µL of TSB medium and cultured for three days at 20°C under stable conditions. At the end of the incubation period, the biofilm structures formed in the liquid interphase were evaluated as rigid and fragile [18,19].

In order to determine the amount of cellulose production in the biofilm structures formed in the liquid-air interphase, 500 µL of overnight active cultures were inoculated into 4500 µL of TSB medium containing Calcofluor (20 μg/mL) and incubated at 20°C under stable conditions for three days. At the end of the incubation period, cellulose production was quantitatively evaluated according to the amount of fluorescence under 366 nm UV light [16].

Swimming and swarming motility experiment

In the evaluation of swimming behavior, firstly, clinical isolates were spread on TSA medium and incubated at 37°C overnight. At the end of incubation, a single colony was taken from the agar surface, placed in the center of a 0.3% TSA plate, and incubated at 20°C, 28°C, and 37°C for nine hours. For the determination of swarming behavior, the strains were similarly incubated in TSA medium overnight at 37°C. At the end of incubation, a single colony was taken from the agar surface and placed on a 0.5% TSA medium containing 0.5% glucose. Agar plates were incubated at 20°C, 28°C, and 37°C for nine hours and evaluated. The motility characteristics of clinical isolates were evaluated by measuring the distance from the inoculation zone to the edge of the swimming and swarming zone on agar plates [20].

## Results

Thirteen patient samples from the Ophthalmology Clinic of Ankara Bilkent City Hospital were numbered consecutively. Since bacterial growth was not observed in patient samples numbered 6, 7, and 11, they were excluded from the study and the study was continued with the samples obtained from the remaining 10 patients. *Pseudomonas* spp. was identified in samples numbered 1, 3, and 9, *Staphylococcus* spp. in samples numbered 2, 4, 5, 8, and 12, *Streptococcus *spp. in sample numbered 10 and *Klebsiella *spp. in sample numbered 13 (Table 2).

**Table 2:** Bacteria identified at the genus level in patient samples.

Bacterial genus	Patient samples
Pseudomonas spp.	1, 3, and 9
Staphylococcus spp.	2, 4, 5, 8, and 12
Streptococcus spp.	10
Klebsiella spp.	13

**Figure 1:**
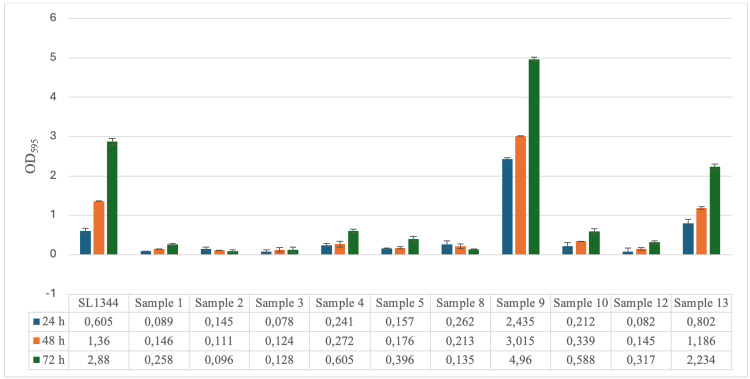
Biofilm production amounts of Salmonella Typhimurium ATCC SL1344 strain and bacteria isolated from patients during 24, 48, and 72 hours at 20°C.

In the biofilm test carried out at 20°C, samples 1, 2, 3, 4, 5, 8, 10, and 12 were considered "weak producers" and SL1344 and samples 9 to 13 were considered 'strong producers' according to the calculation results shown in Table 1. The optical densities at 595 nm wavelength were determined as 2.88 ABS, 4.96 ABS, and 2.234 ABS in samples SL1344, 9, and 13 at 72 hours when biofilm production was the highest (Figure 1).

**Figure 2:**
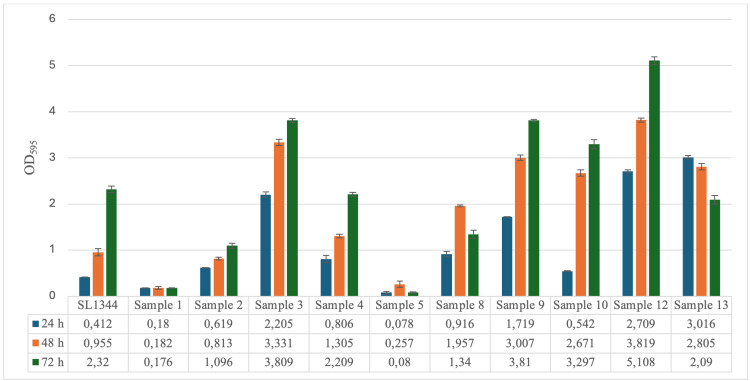
Biofilm production amounts of Salmonella Typhimurium ATCC SL1344 strain and bacteria isolated from patients during 24, 48, and 72 hours at 37°C.

At 37°C, samples 1 and 5 were considered "weak producers," while SL1344 and all remaining samples were considered "strong producers." Among the strong producers, sample 13 reached maximum biofilm production at 24 h with an ABS value of 3,016, while sample 8 reached maximum biofilm production at 48 h with an ABS value of 1,957. Samples 2, 3, 4, 5, 9, and 10, on the other hand, continued to increase their biofilm production at 72 hours, giving results of 1,096 ABS, 3,809 ABS, 2,209 ABS, 3,81 ABS, 3,297 ABS, 5,108 ABS, respectively (Figure 2).

**Figure 3:**
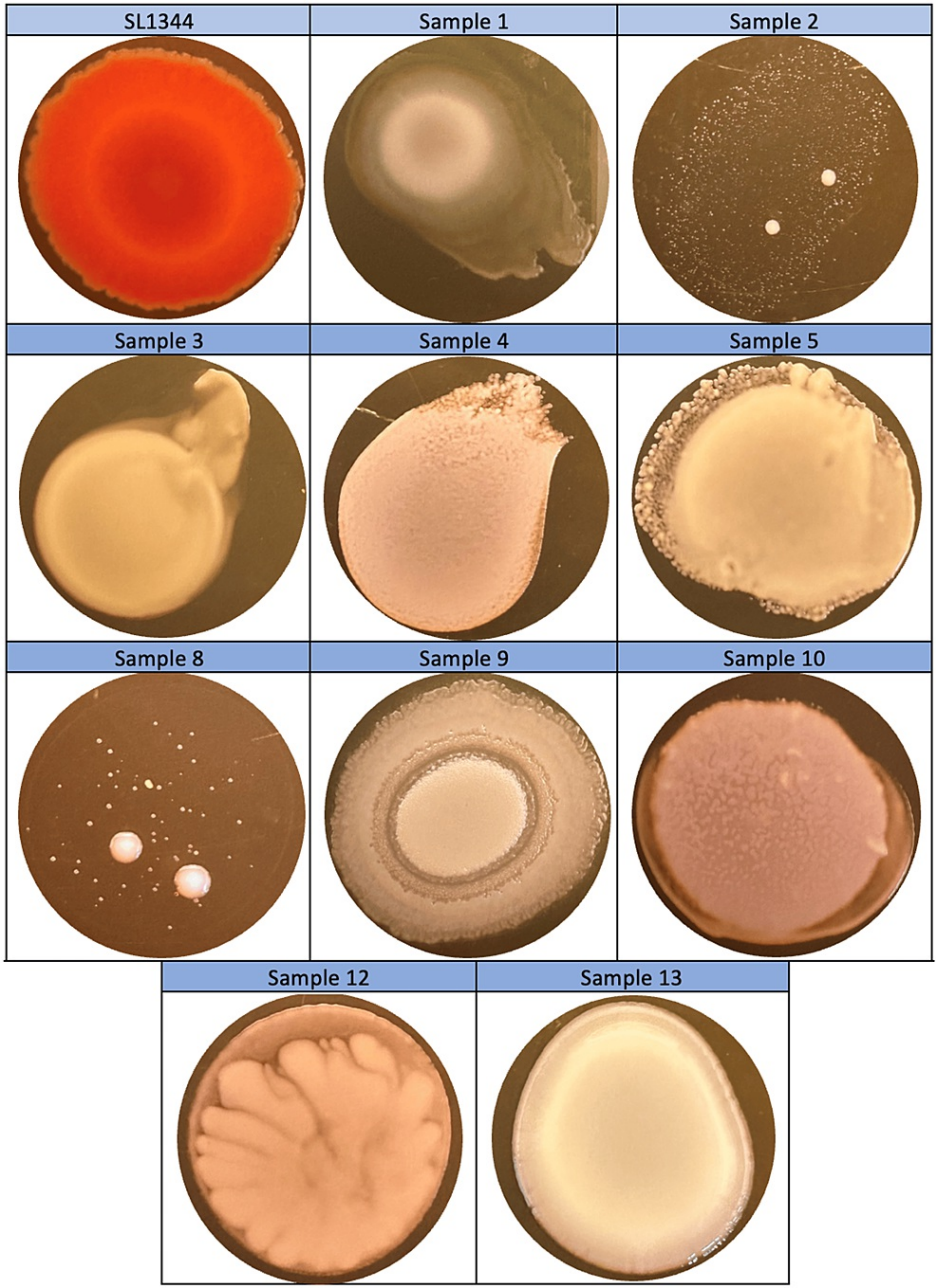
Three-day biofilm morphotypes of Salmonella Typhimurium ATCC SL1344 strain and bacteria isolated from patients in TSA medium containing Congo red at 20°C. TSA: Tryptic Soy Agar.

In the study in which biofilm morphotypes were determined, SL1344 biofilm morphotypes were detected rdar (red, dry, and rough). *Staphylococcus* spp. biofilm morphotypes were found as small colonies with a white center and slime around (samples 2 and 8) and pink, amorphous, cottony, and slime around (samples 4, 5, and 12). *Pseudomonas* spp. biofilm morphotypes were white with a white center, slime around and amorphous structure (sample number 1), white, concave, cottony, and dispersing structure (sample 3), and pink, cottony, containing three phases with different diameters and two different slime layers between these phases (sample 9). *Streptococcus* spp. biofilm morphotype was identified as pink, amorphous, and cottony in the center with a slime zone around it (sample 10). *Klebsiella* spp. biofilm morphotype was identified as white centered, surrounded by slime and shiny structure (sample number 13) (Figure 3).

**Figure 4:**
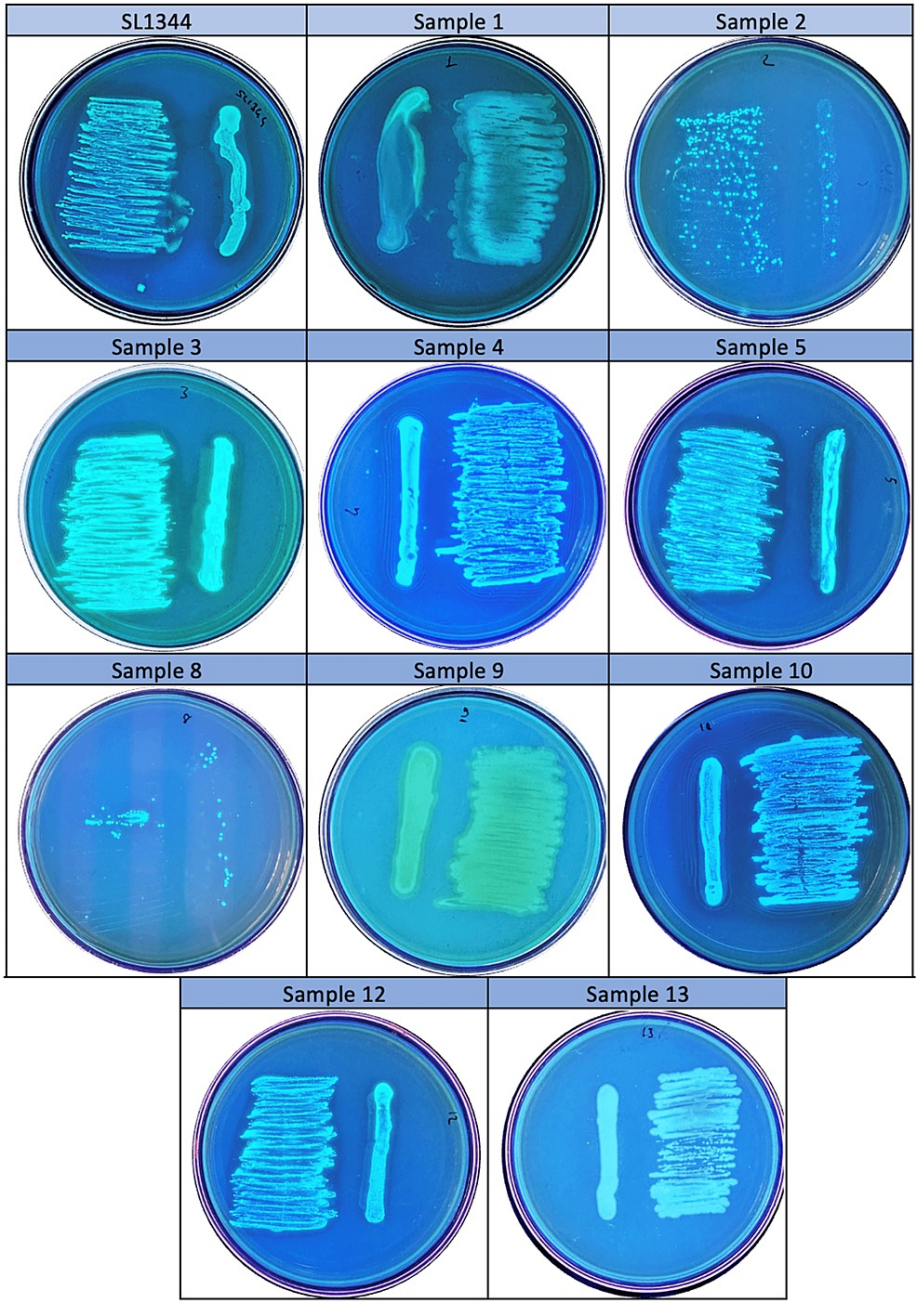
Fluorescence under ultraviolet light of three-day-old biofilm structures of Salmonella Typhimurium ATCC SL1344 strain and bacteria isolated from patients in TSA containing calcofluor at 20°C. TSA: Tryptic Soy Agar.

In the cellulose production capacity study, all clinical isolates cultured on media containing chalcofluor, a cellulose marker, were found to be positive for cellulose production under 366 nm UV light (Figure 4).

**Figure 5:**
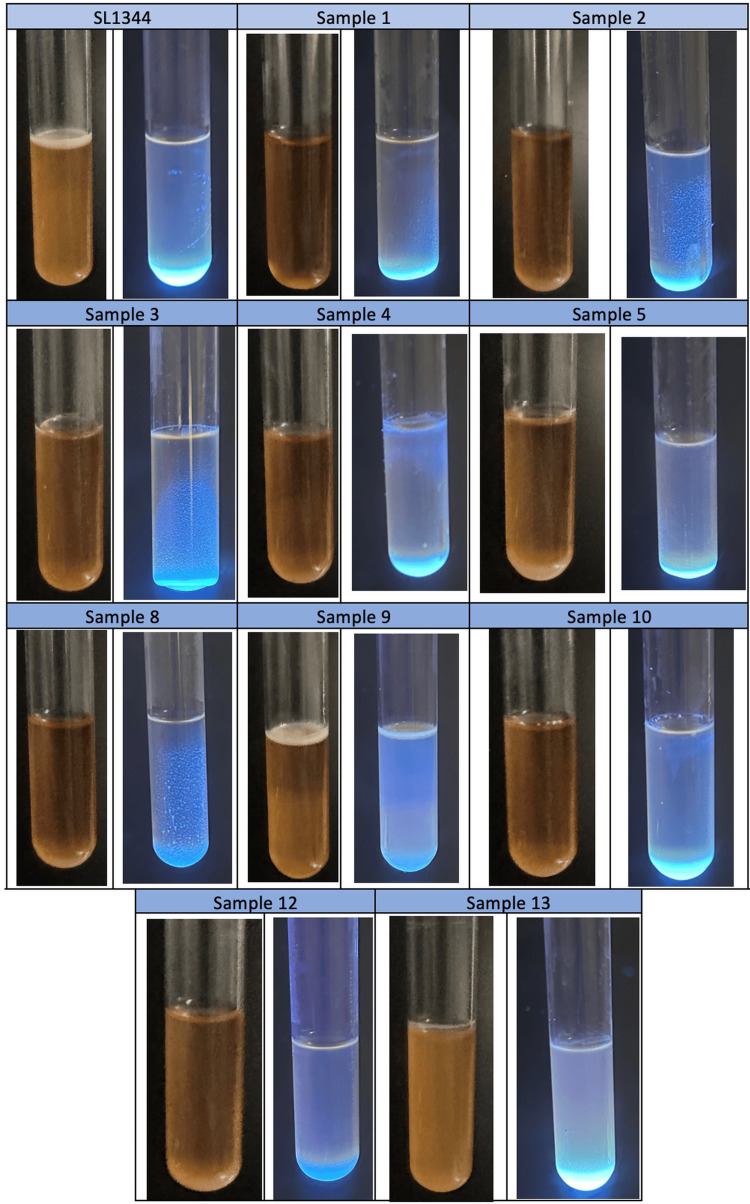
The pellicle structure formed by Salmonella Typhimurium ATCC SL1344 strain and bacteria isolated from patients in the liquid-air interface and the amount of fluorescence.

The higher the production amount of cellulose, which is the main component of the biofilm structure and gives extra strength and elasticity to the bacteria, the resistance of the biofilm structure (pellicle) formed in the liquid-air interphase to mechanical effects such as shaking and mixing increases in direct proportion. While SL1344 and sample number 9, which had the highest pellicle production, formed a "rigid" biofilm which was solid and hard, the other patient samples formed a "fragile" biofilm which was easily dispersed in small amounts. The fact that all patient samples produced on a TSB medium containing chalcofluorine fluoresced under 366 nm UV light indicates that all of them produced cellulose (Figure 5).

**Figure 6:**
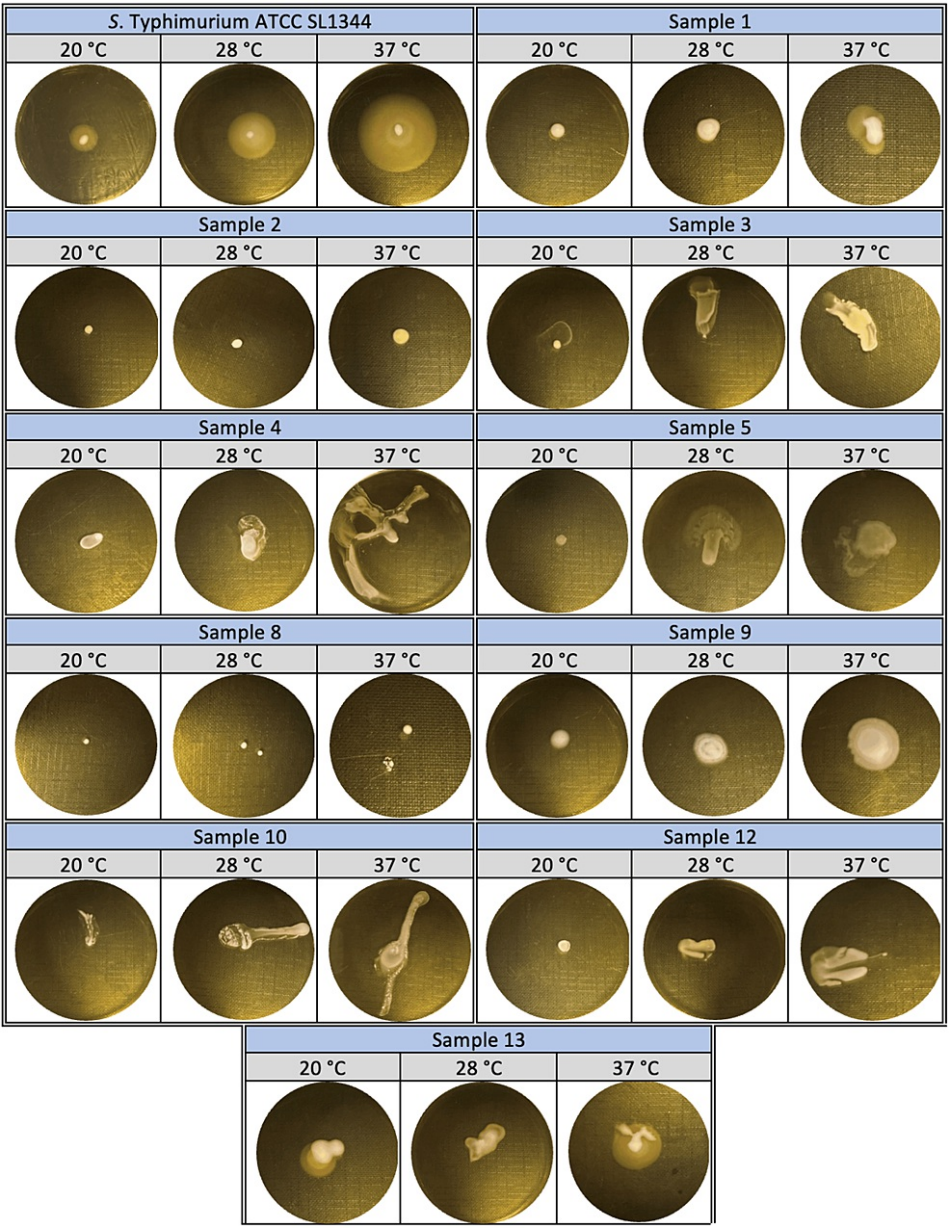
Swimming motility of Salmonella Typhimurium ATCC SL1344 strain and bacteria isolated from patients at 20°C, 28°C, and 37°C.

The swimming motility abilities of SL1344 and samples 1, 5, 9, and 13, which exhibited circular spreading from the center at 37°C, where swimming motility was the highest, were determined as 30 mm, 10 mm, 12 mm, 15 mm, and 13 mm, respectively, by measuring the zone diameters. The mobility distances of samples 3, 4, 10, and 12, which showed a linear spread from the center, were determined as 30 mm, 60 mm, 60 mm, and 30 mm, respectively. In samples 2 and 8, no swimming motility was observed at all temperatures (20°C, 28°C, and 37°C) (Figure 6).

**Figure 7:**
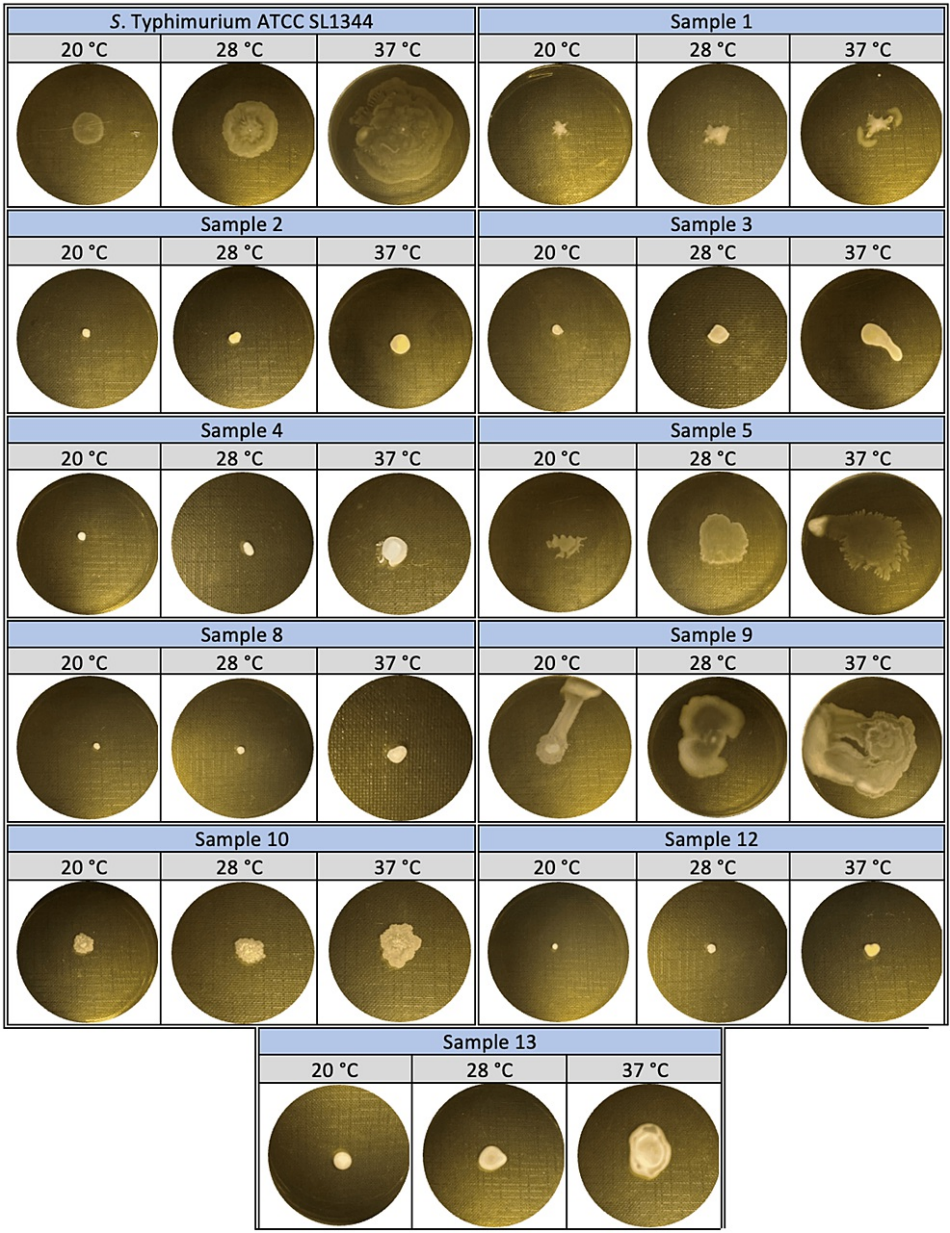
Swarming motility of Salmonella Typhimurium ATCC SL1344 strain and bacteria isolated from patients at 20°C, 28°C, and 37°C.

In the study where swarming motility abilities were determined, 37°C was the temperature with the highest motility. According to these results, the highest swarming motility ability at 37°C was determined in SL1344 (45 mm), sample 9 (40 mm), and sample 5 (38 mm). It was 15 mm in samples 1, 3, and 10; 10 mm in sample 4; and 18 mm in sample 13. Samples 2, 8, and 12 were found to have no swarming motility ability (Figure 7).

## Discussion

Biofilms are now recognized as potential contributors to the development of chronic inflammatory and infectious diseases [13].

Numerous prosthetic device-associated ocular infections have been attributed to biofilm formation on biomaterials implanted in the eyes. These include infections associated with scleral buckles, intraocular lenses, keratoprostheses, and glaucoma drainage implants [8,21-24].

Previous studies have demonstrated biofilm formation on extubated stents from patients, regardless of whether they presented with infection or not [25,26].

When reviewing the literature regarding surgical failure and biofilm formation on silicone tubes, it has been observed that biofilm could potentially contribute to surgical failure [27]. Additionally, biofilm formation on polyurethane nasolacrimal stents has been associated with delayed device failure [28].

In a study aimed at determining the rates of biofilm colonization on silicone stents inserted during dacryocystorhinostomy, it was found that 90% of stent fragments removed eight weeks after surgery exhibited the presence of coccoid and/or rod-shaped bacteria encased in a biofilm matrix [11]. Kim et al. have reported a significant association between *Pseudomonas aeruginosa *infection and membranous obstruction of the nasal mucosa, prolonged silicone intubation, and surgical failure [5]. Balikoglu-Yilmaz et al. have documented that *Staphylococcus epidermidis* and *Pseudomonas aeruginosa* are frequently cultured from lacrimal stents [11]. Similarly, Ali et al. have noted that the most prevalent bacterial organisms found on lacrimal stents are *Pseudomonas aeruginosa *and *Staphylococcus aureus* [29]. In another study, it was observed that the *Staphylococcus aureus *group and *Pseudomonas aeruginosa *group formed significantly higher amounts of biofilms compared to the control group. Bacterial species capable of forming biofilms on silicone tubes included Staphylococcus aureus (week three) and *Pseudomonas aeruginosa* (week four) [30].

Similarly, this study demonstrated biofilm colonization on silicone tubes extracted from patients six months after DCR. In this study, biofilm formation attributed to microorganisms mentioned in the literature, including *Staphylococcus *and *Pseudomonas*, was observed, along with biofilm formation by *Streptococcus *and *Klebsiella*. In addition to existing literature, detailed descriptions of the biofilm formation patterns and characteristics of all these bacteria were provided.

One of the main limitations of this study is the small number of cases. Nevertheless, to the best of our knowledge, this study provides comprehensive insights into the patterns and characteristics of biofilm formation in silicone tubes removed from patients who underwent DCR surgery.

Despite the absence of signs of infection in any of the cases and the surgical success observed in all cases, biofilm formation was detected in patient samples. The moderate to strong biofilm formation characteristics exhibited by the bacteria suggest that these microorganisms could potentially serve as persistent infectious agents.

## Conclusions

Biofilms are considered potential factors in the pathogenesis of infectious and inflammatory diseases. The presence of biofilms in the lacrimal tubes, even if they do not lead to clinical infection, is a broad topic that warrants investigation. In our study, although there were no clinical infections in any of the patients, biofilm formation was detected in the patient samples. The moderate to strong biofilm formation characteristics exhibited by the bacteria suggest that these microorganisms could potentially serve as persistent infectious agents.
